# The Medical Colleges—The Winter Session of Lectures

**Published:** 1888-09

**Authors:** 


BUFFALO MEDICAL AND SURGICAL JOURNAL

A MONTHLY REVIEW OF MEDICINE AND SURGERY.

EDITORS:

THOS. LOTHROP, M. D., - - - W. W. POTTER, M. D.

All communications, whether of a literary or business character, should be addressed to the editors, 284 FRANKLIN STREET, BUFFALO, N. Y.

### Editorial.

### *THE MEDICAL COLLEGES—THE WINTER SESSION OF LECTURES.*

During the present month the annual session of medical lectures in the medical colleges in this city will again open. The Medical Department of Niagara University commences its course on Wednesday, September 19th. The Buffalo Medical College commences Monday, September 24th. The influx of medical students will be observed, and among them the choice of schools, the important question for decision. The advantages offered in Buffalo for the prosecution of work in medical science are surpassed by few, if any, of the inland cities of the country, and its clinical advantages are increasing with wonderful rapidity with each year. The growth of our city within the last decade has been a marvel of American activity, and with our industrial and commercial progress, the clinical facilities for the student in medicine have grown apace. We are able, therefore, to invite the attention of the profession to the unequaled advantages we are able to offer.

With the sentiment now uppermost in the minds of the profession in favor of higher medical education, the duty is imposed upon the colleges to raise their standard, and to place themselves in the vanguard of this onward movement. It must be acknowledged that the Niagara has been placed even in advance of many of the old schools of the State in its compulsory three-years' course, its preliminary examination of the educational fitness of all matriculates, and its graded course of study, with rigid examinations for promotion to its higher classes, and an independent board of examiners, before which the student is required to present himself before receiving his degree. Recent changes in other colleges, especially the College of Physicians and Surgeons of New York, in which this system has been adopted to go into operation with the coming session, demonstrates that the example, boldly and bravely inaugurated five years ago by the young and vigorous school of our city, is followed, even at this early date, by the older and well-established schools. It is quite time for the Buffalo Medical College to swing into line, reform its methods of instruction, and manage its work more for the interests of the profession than, as now, for the pecuniary profit of its faculty. We note with pleasure that it has extended its course to six months. Let it make a three-years' course compulsory, establish a graded curriculum, a board of examiners which shall be something more than an apology for independence in its judgment of the fitness of candidates for graduation.

These criticisms have in view the attainment of an important object, the value of the degree of Doctor of Medicine in this country. Through the mercenary motives actuating the colleges, the profession has suffered until it is only the individual merit of the possessor that saves our degrees from ignominy. Let the standard be so high that, like the universities in England and on the Continent, the holder of the degree shall be honored wherever he may be located.

